# Rate-limiting steps in the dark-to-light transition of Photosystem II - revealed by chlorophyll-*a* fluorescence induction

**DOI:** 10.1038/s41598-018-21195-2

**Published:** 2018-02-09

**Authors:** Melinda Magyar, Gábor Sipka, László Kovács, Bettina Ughy, Qingjun Zhu, Guangye Han, Vladimír Špunda, Petar H. Lambrev, Jian-Ren Shen, Győző Garab

**Affiliations:** 1 Institute of Plant Biology, Biological Research Centre, Hungarian Academy of Sciences, Temesvári körút 62, H-6726 Szeged, Hungary; 2 Photosynthesis Research Center, Key Laboratory of Photobiology, Institute of Botany the Chinese Academy of Sciences, Beijing, 100093 China; 3 Department of Physics, Faculty of Science, University of Ostrava, Chittussiho 10, CZ-710 00 Ostrava, Czech Republic; 4 Global Change Research Institute, Czech Academy of Sciences, Bělidla 986/4a, 603 00 Brno, Czech Republic; 5 Research Institute for Interdisciplinary Science and Graduate School of Natural Science and Technology, Okayama University, 1-1, Naka 3-chome, Tsushima, Okayama 700-8530 Japan

## Abstract

Photosystem II (PSII) catalyses the photoinduced oxygen evolution and, by producing reducing equivalents drives, in concert with PSI, the conversion of carbon dioxide to sugars. Our knowledge about the architecture of the reaction centre (RC) complex and the mechanisms of charge separation and stabilisation is well advanced. However, our understanding of the processes associated with the functioning of RC is incomplete: the photochemical activity of PSII is routinely monitored by chlorophyll-*a* fluorescence induction but the presently available data are not free of controversy. In this work, we examined the nature of gradual fluorescence rise of PSII elicited by trains of single-turnover saturating flashes (STSFs) in the presence of a PSII inhibitor, permitting only one stable charge separation. We show that a substantial part of the fluorescence rise originates from light-induced processes that occur after the stabilisation of charge separation, induced by the first STSF; the temperature-dependent relaxation characteristics suggest the involvement of conformational changes in the additional rise. In experiments using double flashes with variable waiting times (∆τ) between them, we found that no rise could be induced with zero or short ∆τ, the value of which depended on the temperature - revealing a previously unknown rate-limiting step in PSII.

## Introduction

Photosystem II (PSII), or water-plastoquinone oxidoreductase, is a large multi-subunit homodimeric protein complex embedded in the thylakoid membranes of cyanobacteria, algae and vascular plants. The structure of the reaction centre complex (RC) is known at a resolution of 1.9 Å^1^ and our knowledge about the primary and secondary photochemical reactions is also well advanced^2–4^. The electron transfer from the primary donor P680 to pheophytin (Pheo) occurs in several picoseconds; subsequent electron transfer steps on the acceptor and donor sides, respectively - from Pheo^−^ to Q_A_, the first quinone electron acceptor, and from a tyrosine residue (Y_Z_) to P680^+^ - stabilise the charge-separated state. These reactions are followed by electron and proton transfer reactions between the primary and secondary quinone acceptors, Q_A_ and Q_B_ - on the acceptor side, and between Y_Z_ and the S-states of the Mn_4_CaO_5_ cluster, i.e. the oxygen-evolving complex (OEC) - on the donor side.

Recent time-resolved serial femtosecond crystallography experiments on PSII crystals uncovered structural changes accompanying the reactions around the Q_B_/non-heme iron and the Mn_4_CaO_5_ cluster^5^. This raises the question if the variable chlorophyll fluorescence, which is proportional to the quantum efficiency of PSII^6^, contains any component originating from similar conformational changes, as proposed earlier by some authors^7,8^.

The origin of chlorophyll-*a* fluorescence transients has been debated in the past decades and remains controversial. According to the mainstream concept^9,10^, in dark-adapted leaves or thylakoid membranes the multiphasic rise from the minimum to the maximum fluorescence level, *F*_o_-to-*F*_m_, elicited by a long saturating flash or by a series of intense short flashes, reflects solely the reduction of Q_A_. In contrast, some authors have suggested that additional reactions and/or conformational changes must be taken into account^7,8,11–13^. The modified version of the mainstream model^10^ offers explanation on the complexity of the fluorescence induction, the so-called OJIP curve, which contains a wealth of information on the functioning of the photosynthetic apparatus under different experimental conditions^6,10,14–17^. The letters J and I signify intermediate states between the minimum and maximum fluorescence levels, O and P correspond to *F*_o_ and *F*_m_ levels, respectively, with *F*_o_ ascribed to all Q_A_ oxidized and *F*_m_ to all Q_A_ reduced. According to this model “in order to reach [*F*_m_], it is necessary, and sufficient, to have Q_A_ completely reduced in all the active PSII centers”. In sharp contrast to this concept, Vredenberg^12^ has suggested that “full reduction of Q_A_ is neither sufficient nor required for reaching the *F*_m_”; instead, the fluorescence rise is proposed to be “promoted by photo-electrochemical and electrical events in the vicinity of the membrane-bound RC”. Analysis of OJIP transients in a wide temperature-range revealed (an) additional process(es) of different physical origin in the fluorescence rise once Q_A_ is reduced; Schansker and co-workers suggested the involvement of light-induced conformational changes in the variable fluorescence, *F*_v_ = *F*_m_ − *F*_o_^8^.

A basic problem with the mainstream model is that in the presence of DCMU (3-(3′,4′ dichlorophenyl)-1,1′ dimethylurea), which inhibits the electron transfer between Q_A_ and Q_B_, and thus permits only one stable charge separation, *F*_m_ is not reached upon excitation by a single-turnover saturating flash (STSF)^11^. The first STSF induces an intermediary level, hereafter referred to as *F*_1_, typically 75–85% of *F*_m_ and *F*_m_ can be reached only gradually by further excitations^11^, e.g. by several additional STSFs (Supplementary Figure S1). It was shown that, in contrast to the *F*_o_-to-*F*_1_ rise, leading to the reduction of Q_A_, the consecutive flash-induced rises are associated with non-electrogenic reactions - showing their different physical origin. These data have been explained^18^ by assuming the presence of another quencher, different from Q_A_, called Q_2_. However, the identity of Q_2_ has remained elusive.

Another set of data in the literature, which motivated our study, was that the kinetics of the fast fluorescence rise had been shown to depend on the length rather than on the intensity of the excitation flashes^19,20^. With sub nanosecond flashes the rise was purely exponential while with increasingly longer flashes, in the microseconds to milliseconds range, the kinetics became more and more sigmoidal. The authors interpreted their observations within the frameworks of the theory of “interunit transfer of excitations”, i.e. the theory of connectivity between PSII units^21^. However, they noted that “the basic phenomena underlying [the observed] differences should be investigated further”^20^. These data, by taking into account that the shape variations of the rise kinetics were coupled with changes in the *F*_m_/*F*_o_ ratio^20^, could be brought into harmony with the theory of connectivity^22^ but other explanations cannot be ruled out. In fact, it has been proposed that sigmoidicity might arise from overlapping exponential kinetic components^23^. Indeed, Schansker and co-workers^8^, via recording OJIP curves at different, physiological and cryogenic temperatures, established that the overlapping components possessed different temperature dependences and observed the loss of sigmoidicity below −10 °C. (However, a role of connectivity in the sigmoidicity at physiological temperatures could not be ruled out).

With regard to the kinetics of the fast rise, the question can be raised why and how the length of the exciting flash determines both the magnitude (the *F*_m_/*F*_o_ ratio) and the shape of the fluorescence rise (exponential with short flashes and sigmoidal with long ones)^19,20^. A reasonable explanation might be that the long flashes trigger a second process, which then raises the fluorescence yield (increases the *F*_m_/*F*_o_ ratio) and, because of the kinetic overlap with the first process (the reduction of Q_A_), the rise becomes sigmoidal. It is important to see, which also follows from the experiments of Joliot and Joliot^11^, that the second reaction occurs after the first one is completed (“the reduction of C550 attains its maximum level after the first saturating actinic flash…, unlike the fluorescence yield, [for which, i.e. for *F*_m_] several [additional] flashes are required”). Based on these data^11,19,20^, it can be argued that there must be a waiting time between the two types of reactions; in other terms, a rate limitation in PSII – at least as reflected by the reaction(s) leading to the fluorescence rise. Heuristically, (a) rate-limiting step(s) must be involved in the fluorescence rise: we know that the fluorescence yield after the first STSF excitation does not depend on its intensity^19,20^ – the saturation of flashes is routinely checked, e.g. by firing two STSFs simultaneously, which should not affect the fluorescence levels. Nevertheless, to the best of our knowledge, such rate-limiting steps have not been identified and characterized.

The major aim of this work was to address the above two questions, i.e. (i) on the origin of the *F*_1_-to-*F*_m_ fluorescence increment in DCMU-inhibited PSII RC, and (ii) on the basic features of the (previously unidentified) rate-limiting step(s), which is now suggested to determine to a significant extent the kinetics of fluorescence rise. To this end, we investigated the gradual increase of fluorescence yield using STSF excitations on DCMU-treated whole cyanobacterial cells, plant thylakoid membrane preparations and cyanobacterial PSII core particles in a broad temperature range, between about 170 and 300 K. Our data show that in order to reach *F*_m_, it is necessary to have Q_A_ reduced but additional processes succeeding this step, most probably conformational changes, must be involved. Further, by using double-STSF excitations, we show that the fluorescence rise kinetics is largely determined by temperature-dependent rate-limiting reactions in PSII. We propose the involvement of dielectric relaxation processes due to the presence of large static and transient electric fields, generated by the STSF-induced stable and transient charge separations with open and closed reaction centres, respectively.

## Results

### Characterization of the STSF-induced fluorescence rises

In order to discriminate between the *F*_o_-to-*F*_1_ and the *F*_1_-to-*F*_m_ rises and to elucidate the origin of the *F*_1_-to-*F*_m_ increment, we recorded the STSF-induced variations of the fluorescence yield on dark-adapted PSII core particles, isolated from *Thermosynechococcus vulcanus* cells, and isolated spinach thylakoid membranes in the presence of DCMU.

**Figure 1:**
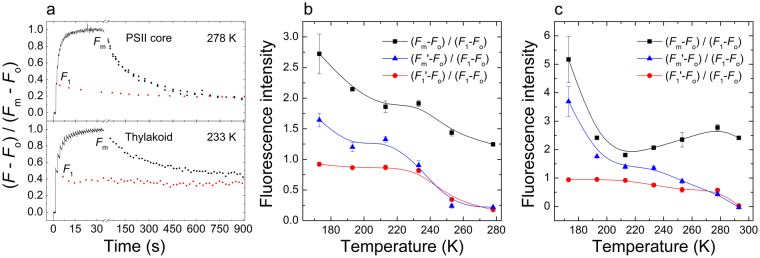
Temperature dependence of chlorophyll-*a* fluorescence induction. Kinetic traces at different temperatures (**a**) and parameters of STSF-induced fluorescence transients as a function of temperature in isolated spinach thylakoid membranes (**b**) and in isolated PSII core complexes of *T*. *vulcanu*s (**c**). In panel a, red and black data points belong to transients induced by a single STSF and a train of STSFs, respectively. *F*_m_′ and *F*_1_′ denote the corresponding fluorescence levels measured after 15 min in the dark following the excitation(s), and thus the black, blue and red curves represent the temperature dependences of *F*_m_, *F*_m_′ and *F*_1_′, respectively, normalized to *F*_1_. In panel a, the decay kinetics of *F*_1_ and *F*_m_ levels were measured on separate samples. In all cases when measuring the decay kinetics, in order to avoid its actinic effect, the measuring beam was turned on and off intermittently.

As shown in Fig. 1a (upper traces), in PSII core at 5 °C (278 K), the first STSF induced a fluorescence increase from *F*_o_ to *F*_1_, to a level which was only about 35% of *F*_m_. An additional train of flashes consisting of 10–12 STSFs was required to reach *F*_m_. At this temperature, which is relatively low compared to the 55 °C growth temperature of cells, charge recombination is limited. Accordingly, during 15 min in darkness *F*_1_ decreased only by about 20%. (Indeed, in thermophilic cyanobacteria, the thermoluminescence band arising from S_2_Q_A_^−^ recombination has been found at 35 °C^24^, indicating a reasonably high stability of the charge separated state at 5 °C). Similar features – large *F*_1_-to-*F*_m_ increment and an almost stable *F*_1_ level – can be observed in spinach thylakoid membranes at cryogenic temperatures (Fig. 1a lower trace). In contrast to the stability of *F*_1_, the *F*_m_ levels decayed considerably more rapidly, confirming that the physical processes determining the two levels differ from each other (see Introduction). Whereas the *F*_o_-to-*F*_1_ transient can be unequivocally assigned to the reduction of Q_A_, the *F*_1_-to-*F*_m_ rise must have a different origin, which remains to be identified. At higher temperatures, 20 °C (293 K) for PSII core and 5 °C (278 K) for thylakoid membranes, the build-up characteristics of the fluorescence yield upon the excitation of the samples with a train of STSFs (Supplementary Figure S2) were qualitatively very similar to those at lower temperatures (Fig. 1a). The main differences were in the decays; in particular, in the relaxation of the *F*_1_ levels, which accelerated with the increase of temperature, evidently due to recombination processes leading to the reoxidation of Q_A_^−^.

### Activation energy associated with the relaxation of the fluorescence increment

In order to separate more clearly the two types of components of the fluorescence rise induced by a train of SFSFs, i.e. the *F*_o_-to-*F*_1_ rise and the *F*_1_-to-*F*_m_ increment, we carried out experiments in a broad temperature range. In particular, we wanted to study the nature of the *F*_1_-to-*F*_m_ increment without the influence of *F*_1_ relaxation. This could be performed in the temperature ranges where *F*_1_ levels exhibited high stability on the timescale of minutes. As shown in Fig. 1b and c, for DCMU-treated thylakoid membranes and PSII core, respectively, in broad, low temperature ranges, *F*_m_ levels decayed at all temperatures (compare black squares with blue triangles), while the *F*_1_ levels remained nearly unchanged (red circles). Decay of *F*_m_ was observed even at 77 K, as checked on a leaf immersed in liquid nitrogen (not shown). The increments, in general, were larger at low temperatures, albeit the temperature dependence did not follow a simple rule in either sample – a phenomenon worth of further investigations. In PSII core, contributions to the increment from PSI are evidently ruled out. For thylakoid membranes, this is excluded by the data obtained with different optical filters, with one of them preferentially allowing the detection of PSII emission, while the other suited both for PSII and PSI emission (Supplementary Figure S3). Hence, we confirmed that *F*_1_-to-*F*_m_ originated solely from PSII.

**Figure 2:**
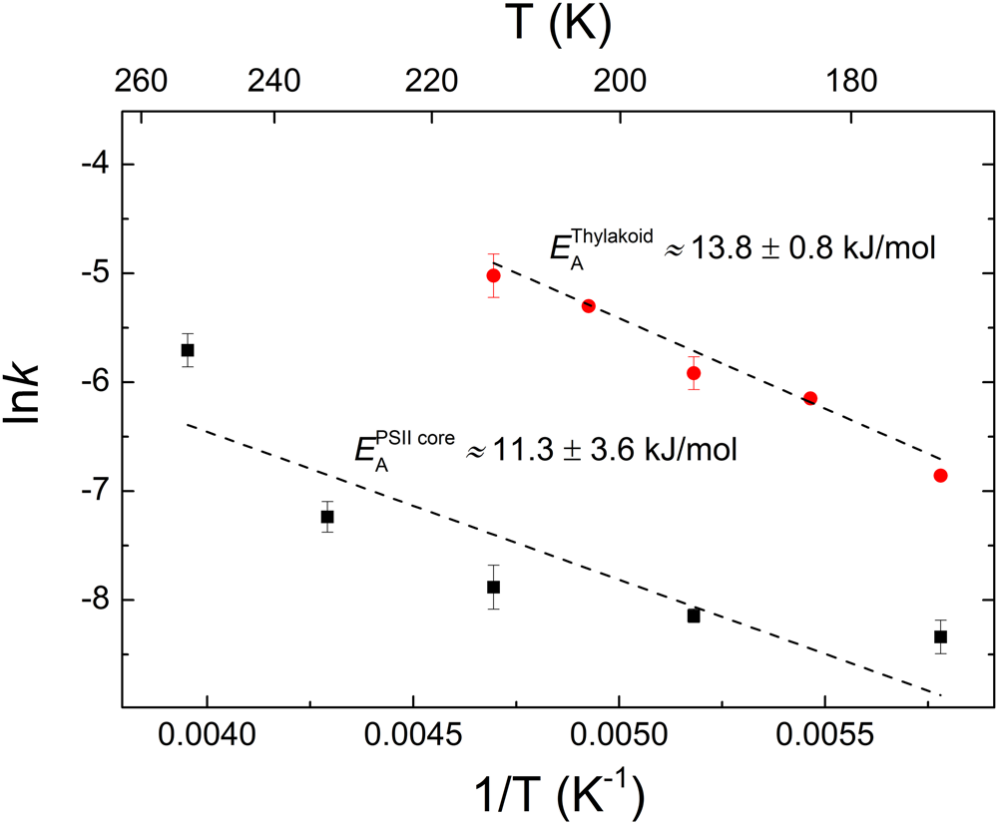
Activation energies associated with the fluorescence relaxation in thylakoid membranes and PSII core particles. The activation energies (*E*_A_) were calculated from the decay of *F*_m_ in a temperature range where the decay of *F*_1_ was negligible.

We analysed the temperature dependence of *F*_m_ decay of thylakoid membranes and PSII core. The analysis was performed on the decay kinetics between about 170 and 210 K, for thylakoids, and about 170 and 250 K, for PSII core. In these ranges, the corresponding *F*_1_ values remained constant and charge recombination involving Q_A_^−^ could be neglected. The calculated activation energies, 13.8 ± 0.8 kJ/mol and 11.3 ± 3.6 kJ/mol, for thylakoid membranes and PSII core, respectively (Fig. 2), agree reasonably well with each other and the values derived from the temperature dependences of the OJIP fluorescence rise components in thylakoid membranes^8^.

### Rate-limiting steps in the reactions associated with the fluorescence increments

**Figure 3:**
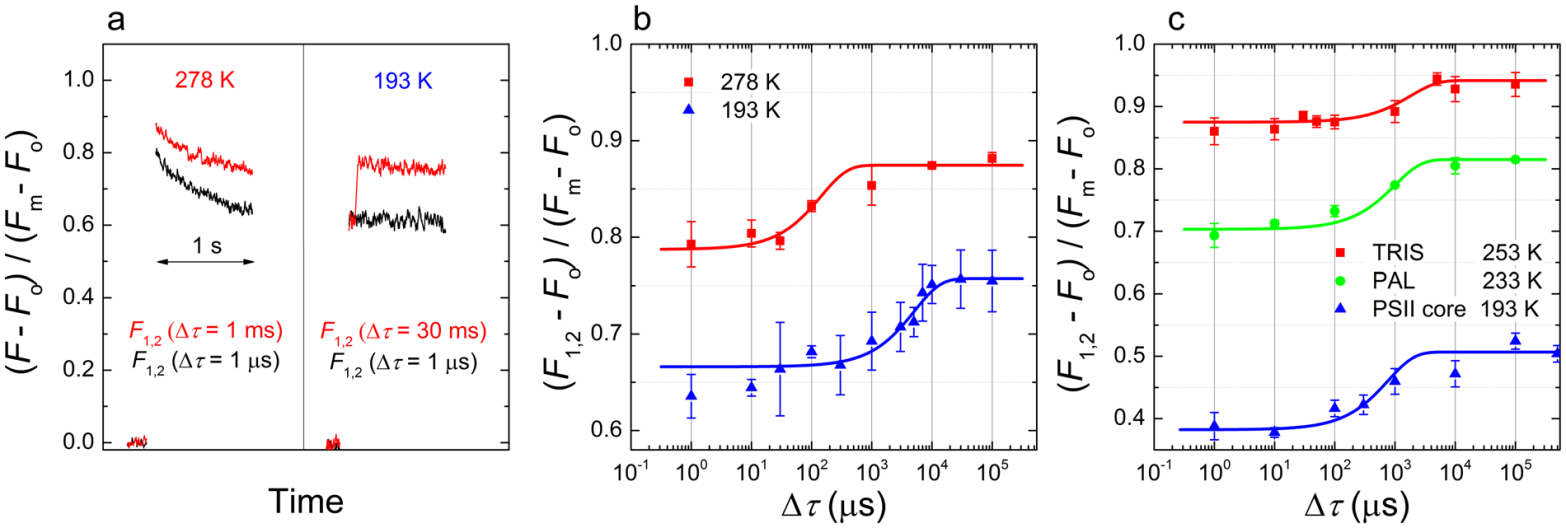
Chlorophyll-*a* fluorescence induced by double flashes. Kinetic traces of the *F*_1_-to-*F*_2_ increment with two different dark times (Δτ) between the first and second flashes, at two different temperatures in isolated spinach thylakoid membranes (**a**) and dependences of the *F*_1_-to-*F*_2_ increments on the waiting time (Δτ) between the flashes in thylakoid membranes (**b**), and in TRIS-washed thylakoid membranes, intact cells of the PAL mutant of *Synechocystis* PCC6803 and in the PSII core particles (**c**) at different temperatures as indicated. *F*_1,2_ denotes the fluorescence intensity level following the first double STSF.

In order to understand the mechanism underlying the variations in the fast fluorescence rise kinetics^20^ (exponential with short pulses and sigmoidal with longer flashes), we performed double-STSF induced fluorescence kinetic measurements. We observed that when the two flashes were fired simultaneously or with a short dark interval between them, no fluorescence increment could be observed (Fig. 3). There was a minimum waiting time (∆τ), which was required after the first STSF, for the second STSF to induce any observable *F*_1_-to-*F*_2_ increment. This suggests that a rate-limiting step is associated with the fluorescence increment. The waiting half-time of the increment was temperature-dependent: in thylakoids it varied between several hundred microseconds at 5 °C (278 K), about 1.3 ms at −10 °C (283 K) (not shown) and almost 10 ms at −80 °C (193 K) (Fig. 3a,b). The existence of these rate limitations could be clearly discerned in all other samples tested: TRIS-washed thylakoid membranes, PSII core particles, intact cyanobacterial cells (the PAL mutant of *Synechocystis* PCC 6803, lacking the phycobilisome antenna^25^) (Fig. 2c). TRIS washing was used to remove the OEC from the PSII supercomplex. Hence, we can rule out the involvement of the Mn_4_CaO_5_ cluster. (This was unlikely, also because in the presence of DCMU the second and consecutive flashes can not induce turnover in the S-states of OEC). Isolation artefacts were ruled out by using intact cyanobacterial cells (the PAL mutant *Synechocystis* sp. PCC6803 – this mutant was used to avoid possible interference and high background emission from this antenna that is anchored to the thylakoid membrane).

The existence of rate-limiting step shows again that the processes induced by the first and the consecutive flashes are of different physical origins. In turn, as pointed out in the Introduction, two independent processes may, at least in part, be responsible for the sigmoidal rise of the fluorescence. In good agreement with this notion, the fluorescence induction kinetics of solubilised PSII core exhibited a sigmoidal rise (Supplementary Figure S4). Evidently, in this sample connectivity between the dimeric PSII supercomplexes can be ruled out (but still allowing intradimer energy transfer between the monomers^26^). Nevertheless, the rise, which also depended on the temperature, could be fitted reasonably well with two exponentials. It is to be noted, however, that for a more rigorous mathematical analysis of this fast fluorescence rise kinetics, and the OJIP curves in general, the rate-limiting step(s) should be taken into account. When fitting with two exponentials the waiting time is ignored, or considered to be negligible relative to the rise times. Also, it implicitly assumes that *F*_m_ can be reached in one additional step, which is not the case. Even with the largest ∆τ, *F*_m_ could not be reached by the second STSF, and the number of STSFs inducing *F*_m_ remained invariant on the mode of excitation – as long as two simultaneously fired STSFs were counted as one and ∆τ was sufficiently long. Further, the rate-limiting step was present not only in *F*_1_-to-*F*_2_ but also between later steps (Supplementary Figure S5) – suggesting similar processes during the train of additional STSFs.

## Discussion

The major aim of this work was to resolve some of the controversies over the origin and kinetics of the chlorophyll-*a* fluorescence induction of PSII, perhaps the most widely used probe of PSII activity. In particular, as argued in the Introduction, in contrast to the expectations based on the ‘mainstream’ model: (i) the fluorescence maximum in DCMU-treated samples cannot be reached by one STSF excitation, and (ii) the fast fluorescence kinetics depends on the length, rather than on the intensity of the flash excitation of PSII. In order to understand the underlying physical mechanisms responsible for these phenomena, which are in conflict with the most widely accepted model, we performed experiments on a variety of different samples and at different, physiological and cryogenic temperatures, in the presence of DCMU, which inhibits the electron transfer between the primary and secondary quinone acceptors, Q_A_ and Q_B_. As will be discussed in more detail below: (i) by using trains of STSFs, we provide irrevocable evidence that the first STSF and the consecutive flashes induce different reactions; (ii) our data, derived from experiments using double-STSF excitations, reveal that the stepwise fluorescence rises after the first STSF do not occur in the absence of sufficiently long waiting times (∆τ) between STSFs – showing the existence of rate-limiting steps, with strong temperature dependence but of presently unidentified nature. As a corollary, we show, also experimentally, that the sigmoidal rise does not require the connectivity between PSII supercomplexes.

### The origin of *F*_1_-to-*F*_m_ fluorescence increment differs from that of the *F*_o_-to-*F*_1_ rise

Our experiments have shown, in agreement with some earlier data (see Introduction) that the underlying physical mechanisms induced by the first STSF and the consecutive flashes, responsible for the *F*_o_-to-*F*_1_ rise and the *F*_1_-to-*F*_m_ increment, respectively, are of different nature. This is most clearly seen at cryogenic temperatures where the charge-separated state is stabilised (Fig. 1). In this region, both in isolated plant thylakoid membranes and cyanobacterial PSII core particles, the *F*_1_ levels remain constant but the *F*_1_-to-*F*_m_ increments display strong, temperature-dependent decay characteristics. Hence, we can conclude that, in accordance with the mainstream model^10^, the reduction of Q_A_ is necessary to account for the fluorescence rise from its minimum to maximum value (*F*_o_-to-*F*_m_). However, in contrast to this model, our data show that it is not a sufficient condition. This conclusion is in perfect agreement with that drawn by Joliot and Joliot^11^, who have shown that, in isolated spinach thylakoid membranes at room temperature, the first STSF induces an intense electric field but the consecutive steps are not electrogenic. Hence, the experiments of Joliot and Joliot, at room temperature, and our data, also at cryogenic temperatures and on PSII core particles, show that the first flash, reducing Q_A_, must be followed by several additional excitations to reach *F*_m_. The length of this process (i.e., the number of excitations involved) depends strongly on the temperature, so does the magnitude of the *F*_1_-to-*F*_m_ increment relative to the *F*_1_ level – which can be quite high (~500% for PSII core at 170 K).

As concerns the origin of *F*_1_-to-*F*_m_ increment, there are essentially two types of possibilities: (i) the additional rise originates from the activity (release) of a quencher different from Q_A_ – called Q_2_^18^ or (ii) the fluorescence yield is modulated via additional light-induced reactions.

### Considerations on the involvement of the hypothetical quencher Q_2_

When taking into account the molecular architecture of PSII RC^1^, we can find candidates for the additional quencher (Q_2_). The non-heme iron, which is situated between Q_A_ and Q_B_ and plays a key role in the protonation of the reduced Q_B_^27^, has been proposed^28^ to be identical with Q_2_. However, this is unlikely to be the case, since the oxidized form of this non-heme iron can easily be reduced by a STSF^29^, and in the presence of DCMU it remains stably reduced^30^ - facts which would be difficult to reconcile with the decay of the fluorescence increment (Fig. 1). The reduction of Q_C_, the third (plasto)quinone found in the crystal structure of PSII from *Thermosynechococcus elongatus*^31^, might be involved in light-induced redox reactions^32^, and thus may act as a quencher. However, Q_C_ is unlikely to be present in our PSII core particles – given the fact that it is not found in the crystal structure of the same preparation^1^. Also, given its position in the quinone exchange cavity^31^, it is unclear how Q_C_ would be reduced in the presence of DCMU and reoxidized under the conditions when Q_A_ remains stably reduced at low temperature (cf. Fig. 1). In a theoretical model, in which “cyt b559 [cytochrome b559] accepts electrons from the reduced primary electron acceptor in PSII, pheophytin, and donates electrons to the oxidized primary electron donor in PSII (P680^+^)”, it has been proposed that “F_m_ increases with an increasing amount of initially reduced cyt b559”^33^. However, direct reduction of the cyt b559 by reduced pheophytin seems highly unlikely because of the relatively large distance between the two molecules^31^. Indeed, in the presence of protonophoric compounds, such as CCCP (carbonylcyanide-m-chlorophenylhydrazon), *F*_v_/*F*_m_ has been shown to increase^34^, and fast photoreduction of the autooxidized cyt b559 has also been observed; however, the STSF-induced photoreduction has been shown to proceed via Q_B_^−^, the semireduced secondary quinone acceptor, a reaction inhibited by DCMU^35^. Taken together, these putative quenchers do not explain our observations. The same holds true for the double-hit trapping model, which hypothesizes that the [PheoQ_A_] acceptor pair acts as a two electron trap^36^. With this pair of acceptor molecules it would be difficult to explain the multi-step *F*_1_-to-*F*_m_ increments observed in our experiments. Also, the slowly-turning over, non-Q_B_ RCs, a fraction of the heterogeneous population of PSII in the thylakoid membranes, generate a stable charge separation upon the first STSF and remain silent for seconds afterwards^37^. The involvement of a presently unidentified, low-quantum-yield photochemical reaction, such as between the tyrosine residues and P680^+^ ^38^, the quenching by P680^+^ ^39^ or the participation of components of inactive branch^40^ cannot be ruled out. However, the product(s) (the quencher) must persist for long time periods (to maintain *F*_1_ constant or the higher levels steps only slowly decaying), minutes at low temperatures; also, its concentration should be largely independent of the intensity of the STSF (a single or double STSF should produce the same amount of quencher); and the release of this putative quencher should be possible to induce by light (to produce the increment) but only after a suitable waiting time. These conditions do not seem to apply to these potential quenchers, components participating in the ultrafast photochemical reactions of the RC.

### The possible role of dielectric relaxation following the charge separations

The fluorescence yield of chlorophyll-*a* can also be modulated by minor variations in the physicochemical environment of the molecules and protein complexes and/or subtle conformational changes of the complexes. Spectral properties and fluorescence lifetimes of different fluorophores can be sensitive to variations in the dielectric (micro)environment. The peak position of the absorption spectra of the lipophylic dye merocyanine 540 has been shown to depend strongly on the dielectric constant of the medium^41^. Similar, albeit less marked dependence has been reported for chlorophyll-*a* and bacteriochlorophyll-*a*; the lifetime (fluorescence yield) of these molecules also depend on the refractive index / dielectric constant of the solvent^42^. Substantial variations in the fluorescence lifetime of chlorophyll-*a* have also been observed in plant light-harvesting protein complex II upon the addition and removal of detergents or varying the lipidic environment of the complexes^43,44^. It is thus proposed that variations in the dielectric microenvironment of RC chlorophyll-*a* can be responsible for the *F*_1_-to-*F*_m_ increment.

The OJIP rise in thylakoid membranes has been shown to be sensitive to the transmembrane electrochemical potential gradient^12,45^, generated by charge separation and consecutive electron and proton transfer steps. While we can not rule out the influence of this factor on the *F*_1_-to-*F*_m_ rise, it is clear that in solubilised PSII core the increments occur in the absence of membrane potential. At the same time, however, the local electric field, due e.g. to (S_2_/Y_Z_)^+^Q_A_^−^, the strength of which can be orders of magnitude higher than that of the uniform transmembrane field, induces a redistribution of ions in the electrolyte surrounding the reaction centre^46^. It is thus reasonable to assume that these events affect the fluorescence yield of chlorophyll-*a*. Its possible interplay with the variations in the dielectric microenvironment - via dielectric relaxation – is discussed below.

The exact physical mechanism of the processes underlying the *F*_1_-to- *F*_m_ increment remains to be elucidated. The increments can most easily be interpreted in terms of light-induced conformational changes^8^ – possessing activation energy values (Fig. 2) similar to those determined earlier^8^ under substantially different experimental conditions. With regard to the nature of conformational changes, we hypothesize that they are coupled to the dielectric relaxation in the RC matrix, which is exposed to the strong local electric field of (S_2_/Y_Z_)^+^Q_A_^−^. Similar mechanism has been proposed to be responsible for the light-induced structural changes in the purple-bacterial RC^47^. We would like to stress that this mechanism does not assume additional quencher molecule(s) but rather relies on modulation of the fluorescence quantum yield via the physicochemical environment of chromophores in the RC complex. The dielectric relaxation is a complex phenomenon even for simple proteins; it may contain different components at different temperatures and hydration conditions^48^. In intrinsic membrane proteins or lipoproteins, inherent heterogeneity of the dielectric matrix may further complicate the situation^49^. Nevertheless, a justifiable assumption, the fine adjustments (components of the dielectric relaxation) in the dielectric matrix are hindered more and more at lower temperatures, explaining the increase of the *F*_1_-to-*F*_m_ increment and the number of excitations required at low temperatures (Fig. 1). Fluorescence and absorbance transient measurements with submicrosecond time resolution should clarify if at ambient temperatures the *F*_o_-to-*F*_1_ fluorescence rise is related solely to the reduction of Q_A_ or also contains an additional component. Excitations in the presence of reduced Q_A_ evidently induce transient charge separations, P^+^Pheo^−^ or Y_Z_^+^Pheo^−^ in the ns-µs time domain. The large transient local electric fields^46^ might induce conformational changes in the proteins and/or rearrangements in the dielectric matrix. As an alternative explanation, the involvement of dissipation-induced heat packages^50^ cannot be ruled out; they might transiently’melt’ the RC matrix and facilitate its dielectric adjustments. In either case, the *F*_1_-to-*F*_m_ increment might be part of the dark-to-light transition of the PSII RC following the reduction of Q_A_. Dark- and light-adapted conformational states have also been proposed for purple bacterial RC^51^.

### Origin of the rate-limiting steps

The physical mechanism of the waiting-time related transient inhibition of the flash-induced reaction responsible for the fluorescence increment (Fig. 3) also remains to be identified but a similar, dielectric-relaxation / conformational-change based mechanism could be at play. It is also to be clarified – by ultrafast absorbance and fluorescence transient measurements - if the rate limitation is at the level of primary photochemistry (e.g. the turnover of P^+^Pheo^−^) or at the level of the state of PSII, a state determining its fluorescence yield. In this latter case, the rate limitation is confined to the propagation of the effect of STSF-induced photoreaction(s) to the site of the fluorophore. It is interesting to note that the waiting times determined in our samples are commensurate with the half-times of the electron transfer between Q_A_ and Q_B_, which also displays strong temperature dependence and is related to protein dynamics^52–54^. It is tempting to speculate that the two mechanisms are harmonized. This may constitute a hitherto unknown photoprotection mechanism, which might be capable of down-regulating the turnover of RC upon excess excitation of PSII.

## Materials and Methods

Thylakoid membranes were isolated from fresh market spinach leaves essentially as described earlier^37^, with minor modifications. Briefly, deveined leaves were homogenized in 20 mM tricine (pH 8.0), 400 mM NaCl, 2 mM MgCl_2_, filtered through a nylon mesh and centrifuged for 7 min at 6000 g. The pellet was resuspended in 20 mM tricine (pH 8.0), 150 mM NaCl and 5 mM MgCl_2_, homogenized and centrifuged for 7 min at 6,000 g. The final pellet was resuspended in 20 mM MES (pH 6.5), 400 mM sucrose, 15 mM NaCl, 5 mM MgCl_2_ and stored, until use, in liquid nitrogen at a concentration of 2–3 mg/ml Chl content. Identical results were obtained with fresh preparations. PSII core complex of *T*. *vulcanus* was isolated as described earlier^55,56^. PAL mutant *Synechocystis* sp. PCC6803 cells^25^ were grown photoautotrophically in BG11 medium supplemented with 5 mM HEPES–NaOH (pH 7.5), and 8 μg/ml chloramphenicol. Cells were grown at 30 °C, under continuous illumination at the intensity of 30 μmol photons m^−2^ s^−1^. Cultures were aerated on a gyratory shaker operating at 100 rpm. TRIS washed thylakoid membranes were prepared by the following protocol. The thylakoid membranes were incubated in 1 M TRIS buffer (pH 8.0) for 30 min in complete darkness and shaken from time to time. The suspension was then centrifuged for 7 min at 6,000 g. The pellet was resuspended in 20 mM MES (pH 6.5), 400 mM sucrose, 15 mM NaCl, 5 mM MgCl_2_ and the sample was used freshly.

Relative fluorescence yields were measured using a PAM 101 fluorometer (Walz, Effeltrich, Germany). The frequency of the modulated measuring light (low intensity, non-actinic) was 1.6 kHz. Variable fluorescence was induced by 0.5 J single-turnover saturating Xe flashes (General Radio 1539-A, USA) of 3 µs duration at half-peak intensity. The sample was placed at the sample holder of a thermoluminescence apparatus in order to control the temperature. The timing of the flashes was controlled by using a home-designed programmable digital pulse generator. The decays of each measurements were recorded by using NI DAQ 6001 via custom-designed LabVIEW software. Least-squares optimization was used to estimate the decay parameters. The optimization algorithms were implemented in Matlab (The MathWorks, Natick, MA). For chlorophyll-*a* fluorescence transient measurements the chlorophyll concentration of the thylakoid membranes were diluted to ~100 μg/ml in 20 mM MES (pH 6.5), 400 mM sucrose, 15 mM NaCl, 5 mM MgCl_2_; the PSII core complexes to ~40 μg/ml in 30 mM MES (pH 6.0), 20 mM NaCl, 3 mM CaCl_2_ and 5% glycerol; the PAL mutants to ~50 μg/ml in BG11 medium. DCMU was dissolved in dimethyl sulfoxide (DMSO) and added to all samples immediately before the fluorescence measurements at a final concentration of 40 μM (the final concentration of DMSO did not exceed 1%). Before the measurements, the samples were dark adapted for 5 min at room temperature.

### Data Availability

The datasets generated during and/or analysed during the current study are available from the corresponding author on reasonable request.

## Electronic supplementary material

Supplementary information
