# Supplementary material for: Rate-limiting steps in the dark-to-light transition of Photosystem II - revealed by chlorophyll-a fluorescence induction
Source: Sci Rep. 2018 Feb 9;8:2755. doi: 10.1038/s41598-018-21195-2 (PMC5807364; doi:10.1038/s41598-018-21195-2)
Supplement: Supplementary file 1 — Supplementary information [file 41598_2018_21195_MOESM1_ESM.pdf]

Rate-limiting steps in the dark-to-light transition of Photosystem II - revealed by chlorophyll-*a* fluorescence induction

Melinda Magyar[1], Gábor Sipka[1], László Kovács[1], Bettina Ughy[1], Qingjun Zhu[2], Guangye Han[2], Vladimír Špunda[3,4], Petar H. Lambrev[1], Jian-Ren Shen[2,5], Győző Garab*[1,3]

[1]Institute of Plant Biology, Biological Research Center, Hungarian Academy of Sciences, Temesvári körút 62, H-6726 Szeged, Hungary

[2]Photosynthesis Research Center, Key Laboratory of Photobiology, Institute of Botany, the Chinese Academy of Sciences, Beijing 100093, China

[3]Department of Physics, Faculty of Science, University of Ostrava, Chittussiho 10, CZ-710 00 Ostrava, Czech Republic

[4]Global Change Research Institute, Czech Academy of Sciences, Bělidla 986/4a, 603 00 Brno, Czech Republic

[5]Research Institute for Interdisciplinary Science and Graduate School of Natural Science and Technology, Okayama University, 1-1, Naka 3-chome, Tsushima, Okayama 700-8530, Japan

*corresponding author

Supplementary information

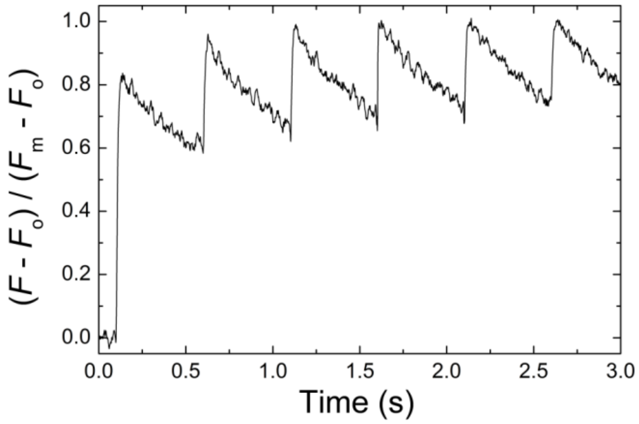

Supplementary Figure S1. **F_o-to-F_m rise induced by a train of STSFs**. Kinetics of chlorophyll-*a* fluorescence of isolated spinach thylakoid membranes in the presence of 40 µM DCMU at 293 K.

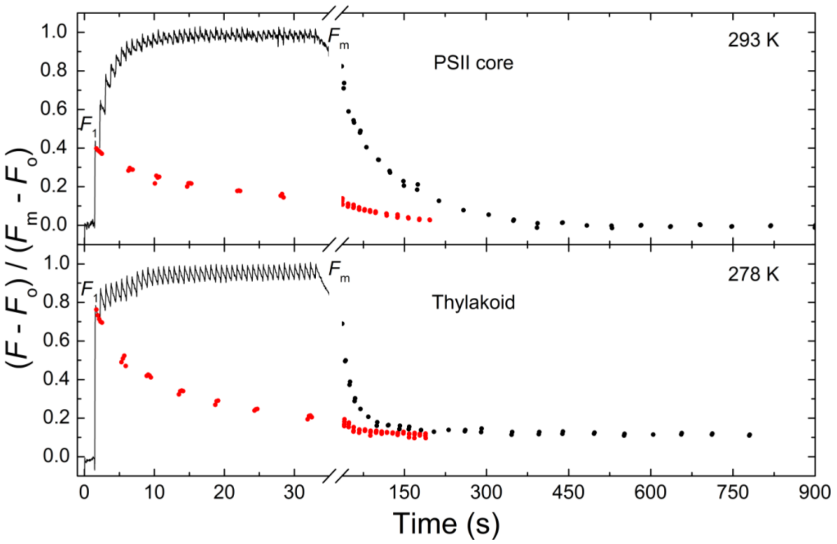

Supplementary Figure S2. Chlorophyll-*a* fluorescence induction and decay at physiological temperatures. Kinetic traces of STSF-induced fluorescence transients in isolated PSII core complexes and spinach thylakoid membranes, measured at 293 and 278 K, respectively. Red and black data points belong to transients induced by a single STSF and a train of STSFs, respectively. As in Figure 1, the measuring beam during the decay was turned on and off intermittently to avoid its actinic effect.

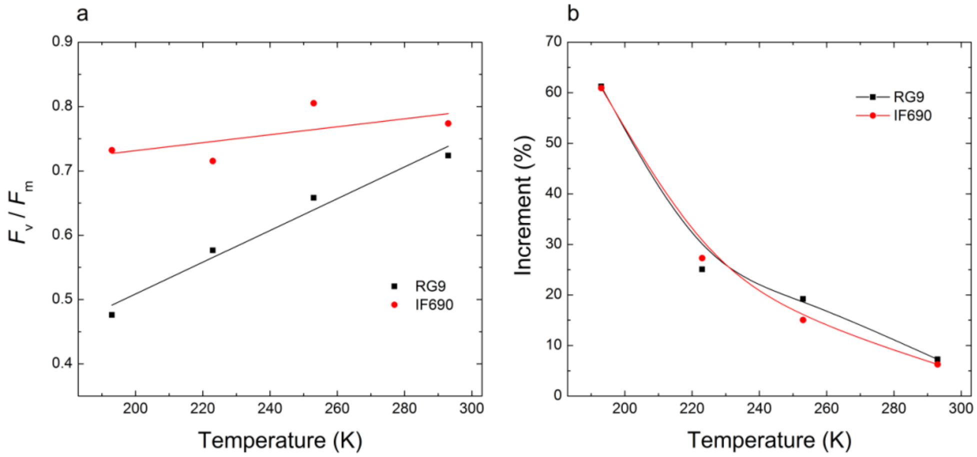

Supplementary Figure S3. Temperature dependences of fluorescence induction parameters recorded with two different optical filters. The F_v/F_m parameter (a) and the F_1–to-F_m increment relative to F_m–F_o (b) of the fluorescence transients of isolated thylakoid membranes measured with Xe-PAM fluorometer using a longpass filter (RG9 >740 nm, which allows the detection of fluorescence both from PSI and PSII) and a narrow-band interference filter (IF690, 690 nm) for PSII emission. Curves in panel b were obtained by b-spline interpolation.

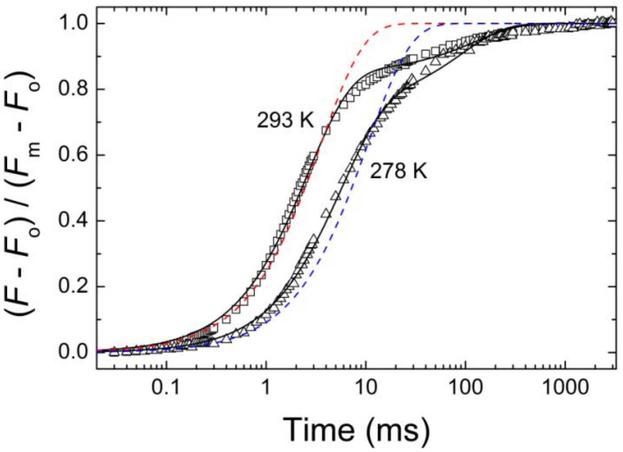

Supplementary Figure S4. Sigmoidicity of the fast fluorescence rise of solubilised PSII core particles. The rise was recorded on DCMU-treated PSII core at 293 and 278 K and the kinetics were fitted with one (dashed lines) and two (continuous lines) exponentials. The measurements were carried out using a Handy-PEA instrument (Hansatech Instruments Ltd., UK), following the method described earlier[8] – using a photon flux density of the excitation of 3500 µmol photons m^{-2} s^{-1}. Fitting parameters: A = 1, $1/k$ = 3.5 ms; A_1 = 0.85, $1/k_1$ = 2.6 ms and A_2 = 0.15, $1/k_2$ = 126 ms (293 K); and A = 1, $1/k$ = 10.4 ms; A_1 = 0.7 5, $1/k_1$ = 5.5 ms and A_2 = 0.25, $1/k_2$ = 92 ms (278 K).

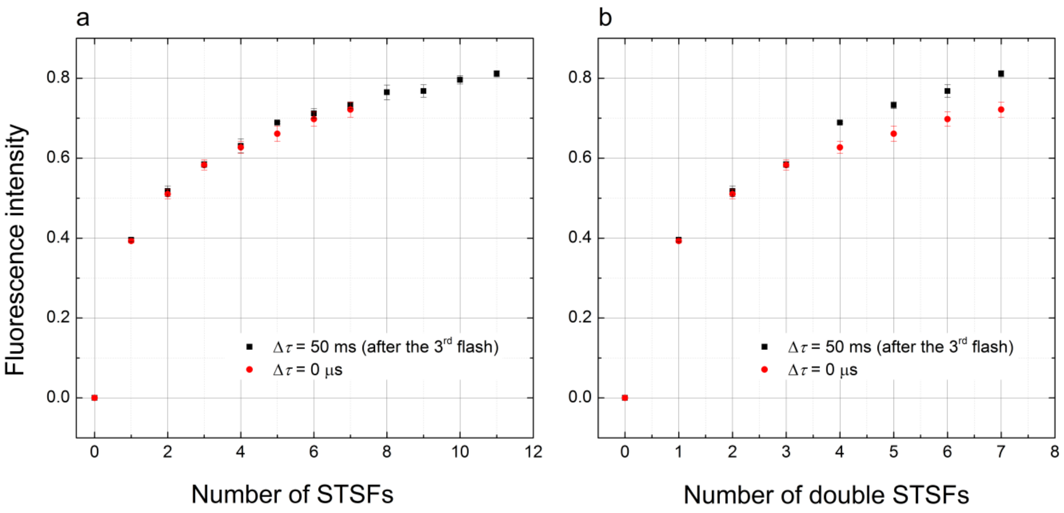

Supplementary Figure S5. Fluorescence intensity levels as a function of the number of flash excitations. Fluorescence intensities, normalized to F_m were measured on DCMU-treated thylakoid membranes at 193 K following two different protocols: (i) double-STSF excitation was applied in the train of seven flashes, with no waiting time between flashes ($\Delta\tau$ = 0 µs, red data points) and (ii) after three simultaneously fired double flashes the waiting time between the two flashes was switched to $\Delta\tau$ = 50 ms (black data points). The obtained data are plotted as a function of the number of STSFs. In panel a, all simultaneously fired flashes are counted as one STSF and the two flashes fired with $\Delta\tau$ = 50 ms are considered as separate STSFs. In panel b, the

fluorescence intensity values are plotted as a function of the number of double flashes irrespective of the value of $\Delta\tau$.
